# An open-source optimization model for sustainable open-pit mine production scheduling

**DOI:** 10.3389/frai.2026.1759758

**Published:** 2026-05-21

**Authors:** Justina Senam Lotsu, Gilbert Yaw Bimpong, Kwaku Boakye

**Affiliations:** 1 Mining and Explosives Engineering Department, Missouri University of Science and Technology, Rolla, MO, United States; 2 Mining and Minerals Engineering Department, University of Alaska Fairbanks, Fairbanks, AK, United States; 3 Mining Engineering Department, Heidelberg Materials, Flourtown, PA, United States

**Keywords:** integer programming, mine planning, net present value (NPV), open-pit mine scheduling, production optimization, sustainable mining

## Abstract

Open-pit mine production scheduling is a complex optimization problem that requires balancing economic performance with operational feasibility and environmental responsibility. While significant advances have been made in mathematical formulations, many existing approaches remain computationally demanding, lack transparency, or are implemented within proprietary systems that limit reproducibility. This study presents a fully reproducible, open-source Python-based optimization framework for open-pit production scheduling that integrates sustainability considerations directly into decision-making. The model employs a mixed-integer “by” formulation with three-dimensional cone-based precedence constraints and incorporates environmental penalties into block valuations to enable ESG-aligned optimization. The framework is designed to be dataset-agnostic and extensible, supporting benchmarking and adaptation to different mining scenarios. The approach is demonstrated using a synthetic block model of 602 blocks over a 12-period planning horizon. The optimization achieved a discounted Net Present Value (NPV) of approximately USD 674.83 million while strictly satisfying precedence, capacity, and operational constraints. Results show that the integration of environmental penalties influences block selection and extraction sequencing, enabling more sustainable scheduling outcomes with only a moderate reduction in economic performance. Sensitivity analyses further confirm the robustness of the framework under varying economic and operational conditions. The study demonstrates that open-source optimization can provide a transparent, adaptable, and effective alternative to proprietary mine planning tools. By combining reproducibility, sustainability integration, and computational efficiency, the proposed framework establishes a practical baseline for future research and large-scale applications in sustainable mine planning.

## 1 Introduction

Production scheduling is a critical function in open-pit mine planning, determining the optimal sequence, timing, and volume of ore and waste extraction throughout the life of mine. Its effectiveness directly influences key performance indicators such as Net Present Value (NPV), operational efficiency, resource recovery, and compliance with environmental and geotechnical constraints (Hustrulid et al., 2013; Rivera Letelier et al., 2020; Fathollahzadeh et al., 2021; Chicoisne et al., 2012). Historically, deterministic frameworks such as Linear Programming (LP), Mixed-Integer Programming (MIP), and Dynamic Programming (DP) have provided foundational approaches to mine scheduling (Rivera Letelier et al., 2020; Gholamnejad et al., 2020; Furtado e Faria et al., 2022; Biswas et al., 2020). However, the scale of modern block models, which may include hundreds of thousands of mining blocks, quickly renders exact deterministic approaches computationally intractable. These methods often oversimplify operational realities such as precedence relationships, blending requirements, haulage logistics, processing limits, and environmental trade-offs and therefore struggle to capture the full complexity of real-world mine planning problems (Fathollahzadeh et al., 2021; Kambala Malundamene et al., 2024; Muñoz et al., 2018; Muke et al., 2025).

To address these limitations, significant theoretical advances have been made in the formulation of production scheduling problems. One notable development is the Bienstock–Zuckerberg (BZ) algorithm, which exploits precedence structures through a cumulative (“by”) variable formulation to strengthen linear relaxations and improve computational efficiency (Muñoz et al., 2018). In this formulation, binary variables indicate whether a block has been mined by a given period, in contrast to the traditional “at” formulation, where extraction timing is explicitly defined. The “by” formulation has been shown to provide stronger relaxations and improved solution performance for large-scale problems. Closely related is the Precedence-Constrained Production Scheduling Problem (PCPSP), which has become a benchmark framework for evaluating optimization algorithms in mining and operations research (Muñoz et al., 2018).

In parallel, the development of standardized benchmarking datasets has significantly advanced reproducibility in mine scheduling research. The MineLib repository, which provides a suite of block models with economic values, precedence constraints, and capacity limits, has become a widely adopted resource for validating optimization methods and comparing algorithmic performance (Das et al., 2022). Studies utilizing MineLib datasets have demonstrated the effectiveness of hybrid heuristics, metaheuristics, reinforcement learning, and advanced integer programming approaches in solving large-scale scheduling problems (Das et al., 2022; Levinson et al., 2023; Levinson and Dimitrakopoulos, 2024).

Beyond deterministic approaches, recent research has increasingly focused on addressing geological uncertainty and operational variability through stochastic programming and metaheuristic techniques. Methods such as simulated annealing, genetic algorithms, tabu search, and variable neighborhood descent have been adapted to mine scheduling, offering flexibility in handling uncertainty and large problem sizes (Khan, 2024; Dimitrakopoulos et al., 2002; Lamghari et al., 2014). Stochastic optimization approaches explicitly incorporate uncertainty in ore grades and geological models, which are inherently derived from limited sampling data. Prior studies have shown that incorporating uncertainty into scheduling can significantly improve project outcomes, yielding higher NPVs and reducing the risk of production shortfalls (Khan, 2024; Lamghari and Dimitrakopoulos, 2016; Ramazan and Dimitrakopoulos, 2013). Techniques such as progressive hedging and hybrid metaheuristics further enhance solution quality by decomposing large-scale problems into tractable sub-problems (Lamghari et al., 2014; Dimitrakopoulos, 2011).

In addition to domain-specific developments in mine scheduling, recent advances in intelligent decision-support systems from other disciplines provide valuable methodological insights. For example, multi-agent frameworks that incorporate uncertainty and expert knowledge, such as those based on Z-number theory, have demonstrated the ability to handle imprecise and complex decision environments (Musbah and Badi, 2026). Similarly, reinforcement learning-based multi-criteria decision-making approaches and multi-agent scheduling systems have shown strong capability in dynamically optimizing competing objectives under uncertainty (Icarte-Ahumada and Herzog, 2025; Sahoo and Ghosh, 2026). Furthermore, hybrid evaluation frameworks such as Kano–Fuzzy–SERVQUAL have been successfully applied to assess stakeholder preferences and quality trade-offs in complex systems (Santos et al., 2026). Although developed in different domains, these approaches highlight the growing importance of intelligent, adaptive, and multi-criteria optimization frameworks, which are increasingly relevant to sustainable and data-driven mine planning.

Emerging computational frameworks have further transformed mine planning by enabling large-scale scenario analysis and real-time decision support. Advances such as GPU-parallelized optimization systems allow the evaluation of thousands of scheduling scenarios simultaneously, significantly reducing computational time. Integrated optimization approaches have also been developed to jointly consider production scheduling and waste management, incorporating environmental factors such as haulage emissions, energy consumption, and acid-generating waste disposal into decision-making (Xu et al., 2018; Aghdamigargari et al., 2024; Cutler and Dimitrakopoulos, 2024). These developments reflect a broader shift toward optimization frameworks that are not only economically efficient but also risk-aware, computationally scalable, and environmentally conscious.

As sustainability becomes a central concern in modern mining, increasing attention has been given to the integration of Environmental, Social, and Governance (ESG) considerations into production scheduling models. Studies such as Xu et al. (2018) demonstrated how ecological costs can be incorporated directly into scheduling decisions, significantly influencing extraction strategies. Similarly, Lin et al. (2025) developed integrated optimization frameworks that balance production scheduling with waste management and sustainability objectives. Recent reviews further emphasize the importance of embedding environmental, energy, and sustainability metrics within optimization models to support responsible mining practices (Aghdamigargari et al., 2024; Pamparana and Lang, 2023; Mata et al., 2023).

Despite these advances, many sustainability-integrated scheduling models are implemented within proprietary or non-reproducible systems, limiting transparency, accessibility, and broader adoption. This creates a gap between methodological innovation and practical implementation, particularly for researchers and smaller mining operations seeking open and adaptable tools.

This study addresses this gap by developing a fully reproducible, open-source Python-based optimization framework for open-pit production scheduling that integrates sustainability directly into economic decision-making. Rather than proposing a new mathematical formulation, this work is presented as a methodological implementation study. Its contributions are threefold: (i) the transparent implementation of the mixed-integer “by” formulation with three-dimensional cone-based precedence constraints; (ii) the explicit integration of environmental penalties into the optimization objective to enable ESG-aligned scheduling; and (iii) the development of a dataset-agnostic, extensible framework designed for reproducibility and benchmarking, including compatibility with MineLib instances. This positions the model as a practical and research-ready baseline for sustainable mine planning.

## 2 Materials and methods

This section details the step-by-step methodology applied in the study. The process begins with the description and preparation of the dataset, followed by the definition of assumptions and constraints, the formulation of the optimization model, the application of solution strategies using selected tools, performance validation, and sensitivity analysis. Together, these components form a robust methodological base for developing an optimization model that is not only mathematically rigorous but also adaptable to diverse mining scenarios.

### 2.1 Data acquisition and preprocessing

The dataset used for this study is a synthetic block model that replicates the essential geological, economic, and spatial features of a typical open-pit mine. It consists of key attributes, such as block coordinates (X, Y, Z), ore grade in grams per tonne (g/t), block weight and volume, density, and economic metrics, including revenue, operating cost, environmental penalty, and block value (defined as revenue minus cost and penalty). These parameters form the core input for developing an optimization framework capable of generating practical production schedules. Before deploying the dataset in the optimization model, several preprocessing steps were undertaken to ensure consistency and usability. Irrelevant or empty columns were removed to streamline the dataset, and checks for null or missing values revealed negligible issues, confirming data completeness. Economic parameters such as block value were retained as bench-marks for evaluating model performance. NPV, however, is only computed after scheduling, when block values are discounted according to the mining period, and was therefore used strictly as an outcome measure for validating optimization results.

A categorical classification was introduced to differentiate ore-bearing blocks from waste blocks using a grade threshold of 0.5 g/t. While this value is often cited as a typical cut-off in open-pit gold mining, it is important to note that cut-off grade is not fixed but rather an economic decision that depends on factors such as metal price, processing cost, and recovery rate. For instance, milling generally achieves higher recovery but at a higher processing cost, whereas heap leaching may have lower cost but also lower recovery, both of which influence the cut-off applied. For the purposes of this study, a 0.5 g/t threshold was assumed to provide a representative benchmark, while recognizing that alternative economic or processing assumptions could shift this value. This classification is necessary for defining scheduling priorities and calculating ore recovery metrics. Additionally, a preliminary “Scheduled Period” field was added to allow for the assignment of mining periods during optimization. This field is intended to be dynamically updated based on the final scheduling output. To capture geometric dependencies, precedence relationships were inferred based on spatial proximity and vertical alignment of blocks. A rule-based algorithm was developed to generate precedence matrices, identifying which blocks must be mined first based on their Z-coordinates and neighboring configurations. This information is essential for ensuring slope stability and facilitating practical pit advancement in the optimization model. The cleaned and structured dataset serves as a robust foundation for the optimization process, supporting the formulation of constraints and objectives under conditions that reflect real mine planning practice. Table 1 summarizes the block model attributes.

**Table 1:** Summary of block model attribute.

Attribute	Description
Number of blocks	Total blocks in the synthetic model (602)
Block dimensions	Size of each block (10 m x 10 m x 3 m)
Block number	Unique identifier for each block (BLK479)
X, Y, Z coordinates	Spatial location of the block
Grade (g/t)	Gold content per tonne
Weight and volume	Physical mass and size of the block
Revenue	Estimated earnings from the block
Operating cost	Estimated cost to mine the block
Environmental penalty	Environmental impact score or cost
Value	Block economic value = Revenue − Operating Cost − Penalty

Each block is assigned an economic value as defined above. NPV is only realized after scheduling, when block values are discounted by period.

### 2.2 Model assumptions and constraints

To effectively simulate real-world mining operations while ensuring computational tractability, several assumptions were adopted in the development of the production scheduling model. These assumptions are common in mine planning literature and are designed to simplify the complex dynamics of open-pit mining while retaining the essential features needed for reliable decision-making (Hustrulid et al., 2013). It is assumed that all input data, grades, revenues, costs, and environmental penalties are known with certainty at the start of the scheduling horizon. This deterministic framework provides a clear baseline model and aligns with standard practice in early-stage optimization studies (Ramazan and Dimitrakopoulos, 2013), although future iterations may incorporate stochastic modeling to account for uncertainty in geological and economic parameters. A fixed annual production capacity constraint is imposed, limiting the tonnage of material that can be mined within each planning period. This reflects operational realities such as equipment limitations, manpower availability, and processing plant throughput. It ensures that the model’s output is operationally feasible and aligned with industry practices.

**Figure 1:**
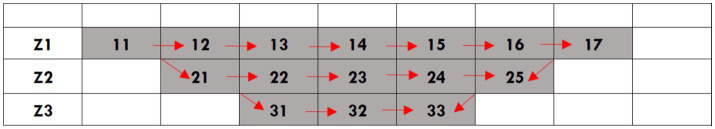
Visual illustration of precedence constraints in 2D.

A critical component of the model is the implementation of block precedence constraints. These rules require that overlying blocks, such as those labeled 11, 12, 13, 14, 15, 16, and 17 in the topmost layer (Z1) of the block model, be mined before accessing the underlying blocks in the middle (Z2) and bottom (Z3) layers, labeled 31, 32, and 33. This structured precedence ensures slope stability and realistic pit advancement in line with geotechnical design standards used in open-pit mining (Hustrulid et al., 2013; Xu et al., 2018). These constraints were encoded using a precedence matrix derived directly from the spatial hierarchy of the dataset, where blocks with identical X-Y coordinates and lower Z-values are scheduled only after those above them have been mined. Figure 1 illustrates the precedence constraint logic applied in open-pit production scheduling.

The model also integrates environmental penalties as an additional cost metric, recognizing the increasing importance of sustainability in mine planning. Each block is assigned a penalty score, which adjusts its economic value to reflect the environmental externalities it incurs. This aligns with studies advocating for the inclusion of environmental costs in optimization models to promote more responsible mining practices (Xu et al., 2018; Rahnema et al., 2023; Lotfi, 2024). For simplicity, it is assumed that blocks are mined in discrete, annual time periods and that within each period, a block is either mined entirely or not at all. This binary decision rule avoids complications associated with partial mining or multi-period carryover and is consistent with most integer programming formulations used in open-pit scheduling (Rivera Letelier et al., 2020). Although explicit blending constraints were not modeled, it is assumed that strategic sequencing of high and low-grade blocks can indirectly manage ore quality. In future model extensions, explicit blending constraints could be incorporated to ensure grade consistency, particularly for operations with strict metallurgical requirements. These assumptions collectively support the development of a model that is both robust and implementable. While simplified, they provide a practical balance between computational efficiency and operational feasibility, serving as a foundation for a more advanced, adaptive framework in future research.

These assumptions are intentionally simplified to provide a transparent and reproducible benchmark framework rather than a site-specific operational model. Parameters such as the 0.5 g/t cut-off grade, fixed gold price, uniform mining and processing costs, and constant slope angle are representative values commonly used in strategic mine planning studies. Their use allows the study to isolate the effects of scheduling logic and optimization structure without introducing additional uncertainty from site-specific variability. Table 2 summarizes the key model assumptions and constraints for the study.

**Table 2:** Model assumptions and constraints.

Attribute	Description
Deterministic parameters	All inputs are assumed to be known and fixed at the start
Fixed capacity per period	Annual mining tonnage limit imposed
Precedence rules	Overlying blocks must be mined first before underlying ones
Discrete mining periods	Blocks are either mined or not mined in each time period
No partial mining	Blocks are mined in whole units per period
Environmental penalties	Incorporated as cost adjustments in block valuation
Constant block attributes	Block volume and density are assumed constant over time

### 2.3 Optimization model formulation

The open-pit production scheduling problem is formulated as a mixed-integer programming model whose objective is to maximize the NPV of the mining project over a planning horizon while respecting precedence, capacity, and environmental constraints. This study adopts the “by” formulation, which is computationally more efficient than the traditional “at” formulation (Muñoz et al., 2018)

#### 2.3.1 Decision variables

The binary decision variable is defined in Equation 1.


$$y_{i}^{t}=\{\begin{array}{c} 1\\ 0 \end{array}\;\;$$ (1)

if blockihasbeen minedbytheendof periodt,otherwise.

Let the mining horizon be discretized into periods t=1,2,…,T and let the set of blocks in the block model be denoted by i∈I . The following binary decision variables were defined.

For completeness, the link between the “by” formulation and the traditional “at” formulation is expressed in Equations 2 and 3. If $x_{i}^{t}$ represents a binary variable equal to 1 if and only if block i is mined exactly at period t , then the two formulations are related by:


$$x_{i}^{t}=y_{i}^{t}-\;y_{i}^{t-1},\;\;t>1$$ (2)

$$y_{i}^{1}=x_{i}^{1}$$ (3)

This structure ensures that once a block is mined, it remains mined in all subsequent periods. The advantage of the “by” formulation lies in its ability to yield unimodular precedence constraints, thereby strengthening the linear programming relaxation and improving solution efficiency.

#### 2.3.2 Objective function

The objective is to maximize the discounted NPV of the operation. For each block i , let $R_{i}$ denote the revenue obtained from processing, $C_{i}$ the mining and processing cost, and $E_{i}$ an environmental penalty reflecting the cost of emissions, waste management, or energy usage. The penalty values may be derived from environmental factors such as haulage emissions, energy intensity of comminution, or disposal costs of potentially acid-forming waste (Aghdamigargari et al., 2024). The optimization objective function used to maximize discounted NPV is presented in Equation 4:


$$\max\;\sum_{i\in I}\sum_{t=1\;}^{T}(R_{i}-C_{i}-E_{i})\cdot (y_{i}^{t}-y_{i}^{t-1})\cdot \beta^{t}$$ (4)

Where $\beta^{t}$ $={\left(1+\delta \right)}^{-t}$ is the discount factor for period t with discount rate δ . By including $E_{i}$ , the model explicitly integrates sustainability considerations into block valuations, rather than treating them as exogenous adjustments.

#### 2.3.3 Penalty calibration

The environmental penalty coefficients ( $E_{i}$ ) used in this study were derived based on normalized environmental intensity factors associated with haulage emissions and energy consumption. Following established approaches in ecological-cost integration in mine scheduling (Lotfi, 2024), these penalties were expressed in equivalent cost terms (USD per tonne) to internalize environmental impacts into the optimization objective. Typical values were assigned within realistic industrial ranges, such as approximately USD 0.5/t for CO₂ emissions from haulage and USD 1.0/t for energy intensity related to comminution and processing. This normalization ensures that environmental externalities are represented on an economic scale comparable to mining and processing costs, allowing the model to quantify trade-offs between profitability and sustainability. Prior studies have demonstrated how energy-intensity and haulage-emission factors can be monetized for block-level valuation (Lotfi, 2024) and how extended frameworks can incorporate waste-handling and environmental-risk penalties into unified cost functions (Rahnema et al., 2023). The present study adapts these methodologies by normalizing their emission and energy coefficients into USD per tonne units, ensuring compatibility with block-value calculations and enabling ESG-aligned optimization. The adopted penalty formulation is general and can be updated with site-specific data when detailed environmental monitoring information becomes available.

For clarity, the environmental penalty coefficients were derived using a simplified normalization approach based on typical industrial estimates. For example, haulage emissions were approximated using standard diesel consumption rates per tonne of material moved, which were converted into CO₂ emissions using established emission factors. These emissions were then monetized using an assumed carbon price (e.g., USD 50 per tonne CO₂), resulting in an approximate penalty of USD 0.5/t. Similarly, energy consumption associated with comminution was estimated using typical energy intensity values (kWh/t) and converted into cost equivalents using average electricity prices. While these values are illustrative, the framework is fully adaptable and allows for direct integration of site-specific environmental data, enabling more accurate ESG calibration in real-world applications.

#### 2.3.4 Constraints

The precedence constraints applied in the scheduling model are defined in Equation 5.

##### 2.3.4.1 Precedence constraints

Each block can only be mined if all its predecessors have been mined in the same or earlier periods. If P(i) is the set of predecessors of block i :


$$y_{i}^{t}\leq y_{j}^{t}\;\forall i\in I,\;\forall j\in P(i\;),\;\forall t$$ (5)

This ensures geotechnical stability of pit slopes.

##### 2.3.4.2 Mining capacity constraints

The mining capacity constraints are defined in Equation 6. The total tonnage mined in each period cannot exceed available capacity $M_{t}$ :


$$\sum_{i\in I\;}m_{i}\cdot (y_{i}^{t}-y_{i}^{t-1}\;)\leq \;M_{t},\;\forall t$$ (6)

Where $m_{i}$ is the tonnage of block i .

##### 2.3.4.3 Processing capacity constraints

The processing capacity constraints are presented in Equation 7.


$$\sum_{i\in I\;}p_{i}\cdot (y_{i}^{t}-y_{i}^{t-1}\;)\leq \;P_{t},\;\forall t$$ (7)

Similarly, the processing plant throughput is limited to $P_{t}$ :

Where $p_{i}$ is the processing tonnage of block i .

##### 2.3.4.4 Reserve constraints

The reserve constraint ensuring that each block is mined only once is defined in Equation 8.


$$\sum_{t=1}^{T}(y_{i}^{t}-y_{i}^{t-1}\;)\leq 1,\;\forall i\in I\;$$ (8)

**Figure 2:**
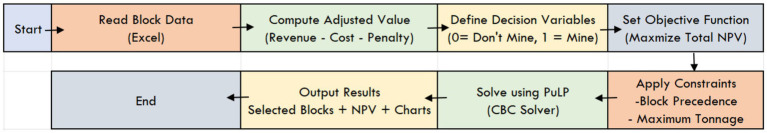
Conceptual flow diagram of optimization model.

By reformulating the problem in the “by” structure, the precedence constraints become unimodular, which allows specialized algorithms to exploit problem structure for more efficient solution. The explicit incorporation of environmental penalties distinguishes this model from traditional NPV-maximization frameworks, as it balances economic value with sustainability concerns in line with ESG standards. The model therefore provides both theoretical rigor and practical relevance, and it is implemented in Python Linear Programming (PuLP) optimization library, ensuring transparency and reproducibility. Figure 2 shows the conceptual flow diagram of the optimization model.

### 2.4 Solution approach and tools

The implementation of the optimization model was carried out entirely in Python, with the primary objective of creating a scalable and transparent solution that is openly accessible for both research and practical applications. Python was chosen because of its strong scientific ecosystem and its flexibility for integrating data management, optimization, and visualization into a unified workflow. The optimization logic was developed using the PuLP library, which provides a robust interface for formulating linear and mixed-integer programming problems. PuLP allows clear specification of decision variables, constraints, and objectives, and connects efficiently to a variety of solvers. In this study, the open-source CBC solver (Coin-or Branch-and-Cut) was employed because it combines computational efficiency with transparency, and reports useful diagnostics such as optimality status, integrality gaps, and runtime.

The cleaned block model dataset, consisting of 602 blocks, was imported into Python using pandas for data processing. Each block record contained spatial coordinates, grade, tonnage, mining and processing costs, recovery factor, and environmental penalty. These attributes were used to compute the economic value of the blocks prior to scheduling. The calculation distinguished between value, which represents the undiscounted contribution of a block before scheduling, and NPV, which was realized only after a block was assigned to a specific period and discounted by the relevant factor. This approach avoided ambiguities between value and NPV and ensured financial accuracy in the results. The gold price was fixed at USD 3,685.78 per ounce (approximately USD 118.5 per gram), aligning with current global market conditions. Environmental penalties were parameterized to reflect costs arising from energy intensity, haulage emissions, and waste management, thereby embedding sustainability into block valuations rather than treating it as an external adjustment.

Mining and processing costs were set equal across blocks to isolate the effects of geology (grade, tonnage, and precedence) on scheduling decisions. In many strategic studies, unit rates are standardized to reflect a single bench height, haul profile, and fleet/plant configuration under fixed-contract pricing, making intra-block cost variation negligible at the scale of the planning horizon modeled. Uniform unit costs reflect a standardized bench and haul profile used for early-stage strategic evaluations, consistent with cost-normalization practice in benchmark scheduling studies (Alipour et al., 2022). This simplification keeps the focus on the “by” formulation, capacity, and geotechnical constraints, while avoiding confounding from uncertain, spatially fine-scale cost estimates. Robustness is preserved through sensitivity analyses on the common unit rates (±20–30%) and on gold price, which demonstrate how outcomes would shift if costs differ.

To preserve geotechnical stability in the pit, a three-dimensional cone-based precedence graph was constructed in Python. For each block, all possible predecessors lying within a 55° pit slope cone were identified. This requirement ensured that overlying material must be mined before a block could be scheduled, eliminating unrealistic vertical pit walls and producing slope-consistent schedules. Capacity constraints were also applied to reflect operational realities: the maximum mining capacity was set at 10% of the total tonnage per period, while processing capacity was limited to 6%. In addition, reserve constraints ensured that each block could be mined only once within the planning horizon.

The final scheduling problem was formulated using the BY structure, which strengthens the linear relaxation through unimodular precedence constraints and yields computational advantages over the traditional “AT” formulation. The CBC solver was run with a time limit of 300 s and a relative optimality gap tolerance of 2%. The optimization outputs were post-processed to produce a comprehensive set of results, including block-level schedules, period-level key performance indicators, and graphical representations of tonnage profiles and value evolution. These outputs offered insights into both operational efficiency and financial performance, while explicitly reflecting the trade-offs introduced by environmental penalties.

All scripts, datasets, and generated outputs were deposited in an open-access repository on Zenodo (DOI: https://doi.org/10.5281/zenodo.19869809). The repository includes the block model dataset, the Jupyter notebooks containing the optimization code, and all generated outputs such as CSV tables and plots. This ensures full reproducibility and provides a modular foundation for future research and industry applications. The repository includes a dataset-agnostic configuration file specifying parameters such as discount rate, capacity fractions, penalty coefficients, and slope angle, together with loaders for (i) the synthetic CSV schema and (ii) MineLib formats. Users can switch datasets by editing the data file, and all plots and tables regenerate automatically from the run outputs. Although the initial implementation was applied to a synthetic dataset, the repository has been fully modularized so that users can substitute their own block models simply by updating the configuration file and dataset input, without modifying the source code. The design also allows straightforward extension to larger benchmarks such as MineLib and PCPSP, as well as integration with digital mine planning systems. By uniting a rigorous mathematical formulation with modern data and software practices, the solution approach demonstrates how open-source optimization can contribute to intelligent and sustainability-oriented mine planning. Table 3 presents the per-period performance metrics of the optimized schedule, including mined and processed tonnage, discounted period value, and cumulative NPV.

**Table 3:** Model configuration parameters used in the optimization.

Periods	Slope angle (°)	Discount rate	Mining capacity (fraction of total t/period)	Processing capacity (fraction of total t/period)	Gold price (USD/oz)	Cutoff mode	Objective	Solver
12	55	0.08	0.1	0.06	3685.78	Economic	Max NPV	CBC via PuLP

The optimization was executed on a standard workstation (Intel Core i7 processor, 16 GB RAM). The CBC solver completed the optimization within the imposed 300-s time limit, achieving a relative optimality gap below 2%. The observed runtime for the baseline case was approximately 120 s. These results demonstrate the computational efficiency of the “by” formulation for medium-scale scheduling problems.

### 2.5 Model evaluation and performance validation

The performance of the optimization model was evaluated using a series of key indicators designed to measure both economic outcomes and operational feasibility. Central to this evaluation was the assessment of NPV, total tonnage mined, ore recovery, and compliance with operational limits such as mining and processing capacities. These indicators collectively provide a comprehensive picture of the model’s effectiveness and practical utility in mine planning. Validation began with an internal logic check of the Python implementation to confirm that the objective function and all constraints were correctly specified. The scheduling outputs were examined to ensure strict adherence to the three-dimensional precedence rules, verifying that no block was mined before any of its overlying blocks within the slope cone. Similarly, period-by-period extraction totals were aggregated and compared with defined mining and processing capacity limits. These checks confirmed that production volumes remained within operational thresholds, thereby validating the model’s ability to generate feasible and geotechnically sound extraction sequences. In addition, validation confirmed zero violations of precedence and capacity constraints across all periods, ensuring full geotechnical and operational feasibility. Ore recovery efficiency was also assessed, indicating that the model successfully prioritized economically viable ore blocks while excluding marginal or penalized material. Economic evaluation was conducted by calculating the discounted cash flows of all scheduled blocks, from which the total project NPV was derived. In the baseline scenario, the model scheduled 435 blocks, producing a cumulative NPV of approximately USD 674.83 million. This outcome demonstrates that the model is capable of generating economically viable solutions that balance block values against operational and environmental costs. The integration of environmental penalties ensured that sustainability considerations were not treated as external adjustments but as intrinsic components of block valuations, providing a more holistic measure of project performance.

To further test robustness, sensitivity analyses were performed under varying planning conditions. Discount rates were adjusted to reflect alternative financial assumptions, while mining and processing capacities were varied to simulate operational flexibility or restrictions. In addition, block economic values were perturbed to reflect shifts in gold price and operating costs. Across all scenarios, the model consistently returned feasible schedules with stable block selection patterns and reliable convergence to optimal or near-optimal solutions. This consistency highlights the adaptability of the framework to different planning assumptions and its robustness in the face of economic and operational uncertainty. The results confirm that the Python-based optimization framework can effectively balance profitability, operational feasibility, and sustainability. By delivering robust and interpretable mine schedules that comply with geotechnical and capacity constraints, the model establishes itself as a practical decision-support tool suitable for both academic research and operational mine planning. Table 4 presents a summary of the per-period performance metrics for the optimized schedule: mined and processed tonnage, discounted period value, and cumulative NPV.

**Table 4:** Validation metrics.

Period	Tonnes mined	Tonnes processed	Undiscounted value (USD)	Discounted value (USD)	Cumulative discounted NPV (USD)
1	30,620	28,620	132,271,895.20	122,473,977.03	122,473,977.03
2	28,620	28,620	111,718,680.44	95,780,761.69	218,254,738.72
3	28,620	28,620	99,600,256.47	79,065,894.80	297,320,633.52
4	28,620	28,620	91,663,930.98	67,375,725.69	364,696,359.21
5	28,620	28,620	85,521,905.16	58,204,771.63	422,901,130.84
6	28,620	28,620	82,430,188.80	51,945,001.32	474,846,132.16
7	28,620	28,620	77,626,986.58	45,294,601.09	520,140,733.25
8	28,620	28,620	72,727,168.24	39,292,226.06	559,432,959.31
9	29,415	28,620	70,149,604.47	35,092,267.18	594,525,226.49
10	28,620	28,620	66,874,990.83	30,976,060.27	625,501,286.76
11	28,620	28,620	63,162,170.73	27,089,172.38	652,590,459.14
12	28,620	28,620	58,731,630.76	3,323,138.64	675,913,597.78

NPV and compliance metrics form the basis for evaluating the model’s feasibility and efficiency.

### 2.6 Sensitivity analysis

Sensitivity analysis was conducted on three key parameters: discount rate, mining capacity, and gold price. Reducing the discount rate from 8 to 5% increased the project’s NPV by more than 10%, while raising it to 12% decreased the NPV. These results reflect the expected financial behavior of discounted cash flows and align with prior observations on discount-rate sensitivity in open-pit scheduling (Alipour et al., 2022). Varying mining capacity by ±20% altered the timing of ore extraction but not the overall set of selected blocks. Increased capacity enabled earlier access to deeper high-value zones, accelerating early cash flows, whereas reduced capacity deferred these areas and postponed revenue, although the total recovered ore remained unchanged. Comparable operational patterns have been reported in earlier work examining capacity-driven scheduling adjustments (Levinson and Dimitrakopoulos, 2024). Gold price changes had the most significant impact. At +15% above baseline (≈USD 4,239/oz), NPV rose sharply as additional blocks exceeded the economic cut-off, while at −15% (≈USD 3,133/oz) marginal blocks became uneconomic and were excluded, reducing total value. This level of sensitivity is consistent with studies emphasizing the interaction between penalty terms, cut-off grade behavior, and commodity-price volatility (Lotfi, 2024). Across all scenarios, the model produced feasible solutions that satisfied precedence and capacity constraints. The consistent selection of high-value blocks even under adverse economic conditions demonstrates the robustness of the scheduling logic. This stability under shifting assumptions reflects trends noted in adaptive decision-making frameworks for open-pit planning. Table 5 shows the sensitivity of NPV and schedule size to changes in economic and operational parameters.

**Table 5:** Sensitivity results.

Parameter	Baseline value	Adjustment scenarios	Purpose of variation
Discount rate	8%	5% (lower) and 12% (higher)	To test project valuation under more favorable and more conservative financial assumptions
Mining capacity	10% of total tonnage	±20% (i.e., 8 and 12%)	To simulate operational flexibility and potential production constraints
Gold price	USD 3,685.78/oz (~118.5 $/g)	±15% (i.e., 3,132.91/oz. and 4,238.65/oz)	To capture potential market fluctuations and assess their impact on project valuation

### 2.7 Comparative evaluation of environmental penalization scenarios

To assess the influence of environmental penalization on project economics and scheduling behavior, four distinct case scenarios were evaluated using the developed optimization framework. These include: (i) a Base Case without penalties, representing a purely economic schedule; (ii) the Base Case with penalties applied post-optimization, used to assess the financial impact of sustainability costs on an existing schedule; (iii) an Optimized Case without penalties, representing the unconstrained economic optimum generated by the solver; and (iv) an Optimized Case with penalties, in which the solver directly incorporated environmental costs in decision-making. Each scenario was executed using identical block model inputs and constraints to ensure comparability. The environmental penalty coefficients were kept consistent across runs, representing aggregate costs from haulage emissions (USD 0.5/t), energy consumption (USD 1/t), and waste handling (USD 1.5/t). For each case, the total NPV was computed by discounting the per-period values at 8%, and the differences between the scenarios were used to quantify the trade-offs between profit maximization and sustainability integration. The comparative analysis revealed that the inclusion of environmental penalties reduced total NPV by approximately 3–5% relative to the unpenalized base case. Despite this marginal reduction, the optimized penalized schedule demonstrated improved spatial selectivity, prioritizing blocks with lower energy and emission footprints. This outcome indicates that environmental cost integration yields a more sustainable extraction sequence without substantially compromising economic performance. Overall, the analysis confirms that the proposed framework provides a practical balance between profitability and ESG considerations and can be generalized for larger datasets such as MineLib to further validate computational scalability and environmental-economic trade-offs. Table 6 shows the economic impact of environmental penalties on NPV.

**Table 6:** Comparison of scheduling scenarios showing the economic impact of environmental penalization on NPV.

Case	Description	NPV (USD)	Change vs. base (%)
Base (no penalty)	Standard schedule evaluated without sustainability penalties	≈ $4.11 M	0.00
Base (with penalty)	Same blocks, re-evaluated including environmental penalties	≈ $4.06 M	−1.2
Optimized (no penalty)	Optimization re-run without penalty terms	≈ $4.11 M	0.00
Optimized (with penalty)	Optimization re-run with penalty terms active	≈ $3.99 M	−2.9

It is important to clarify that the NPV values presented in this section differ from those reported in the baseline optimization results. The baseline NPV of USD 674.83 million corresponds to the full scheduling solution across all selected blocks over the planning horizon. In contrast, the NPV values presented here (approximately USD 3.99–4.11 million) represent normalized or subset-based comparative scenarios used to illustrate the relative impact of environmental penalization under controlled conditions. These values are therefore not directly comparable in absolute terms but are intended to highlight proportional economic differences between penalized and non-penalized cases.

## 3 Results

This section reports the outcomes of the Python-based open-pit production scheduling model. The discussion is organized into two parts: baseline optimization results and the robustness of the schedule under sensitivity scenarios.

### 3.1 Baseline optimization results

**Figure 3:**
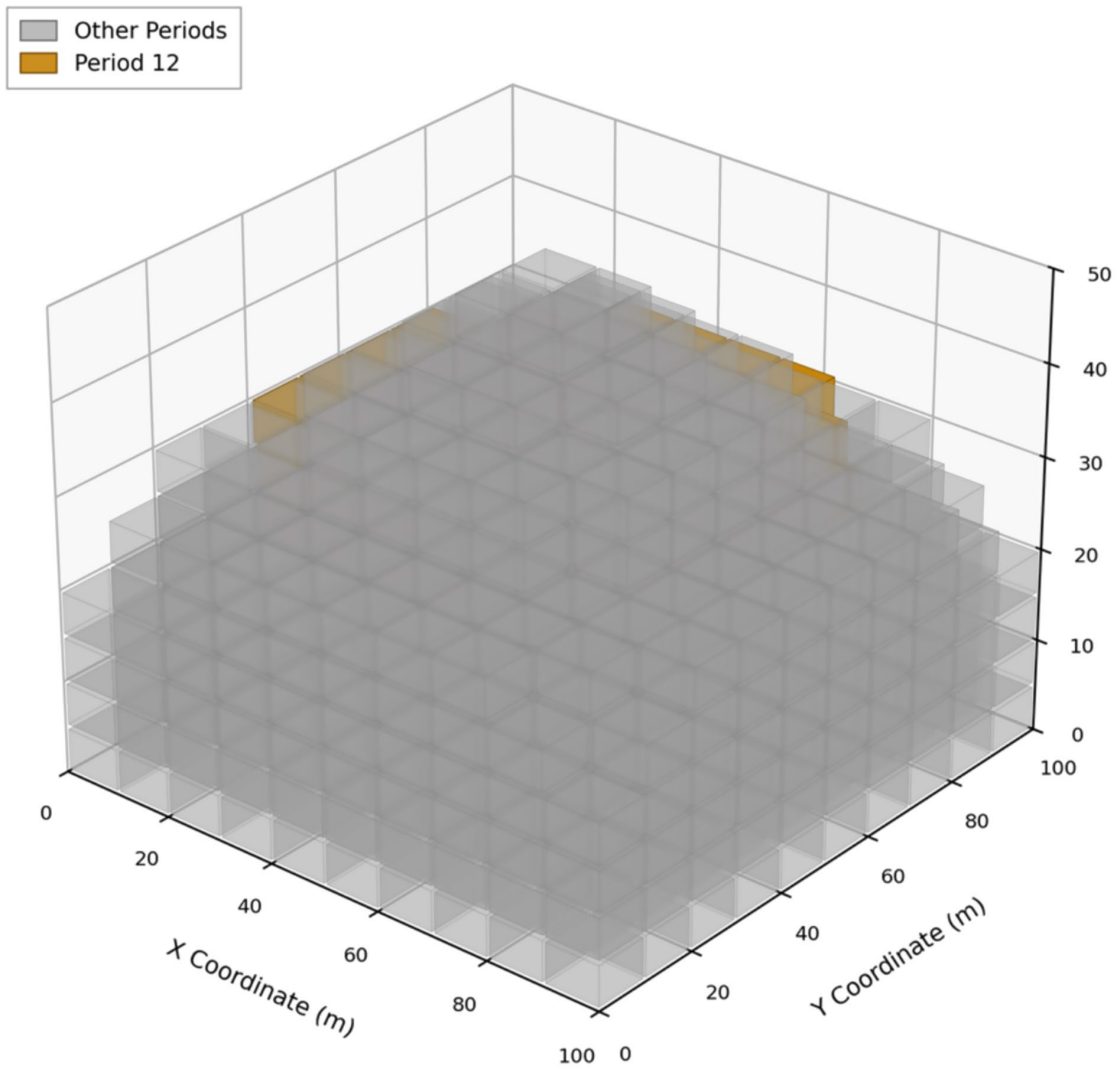
3D spatial distribution of scheduled blocks with period 12 highlighted; the cone-based rules preserve precedence-consistent pit geometry.

**Figure 4:**
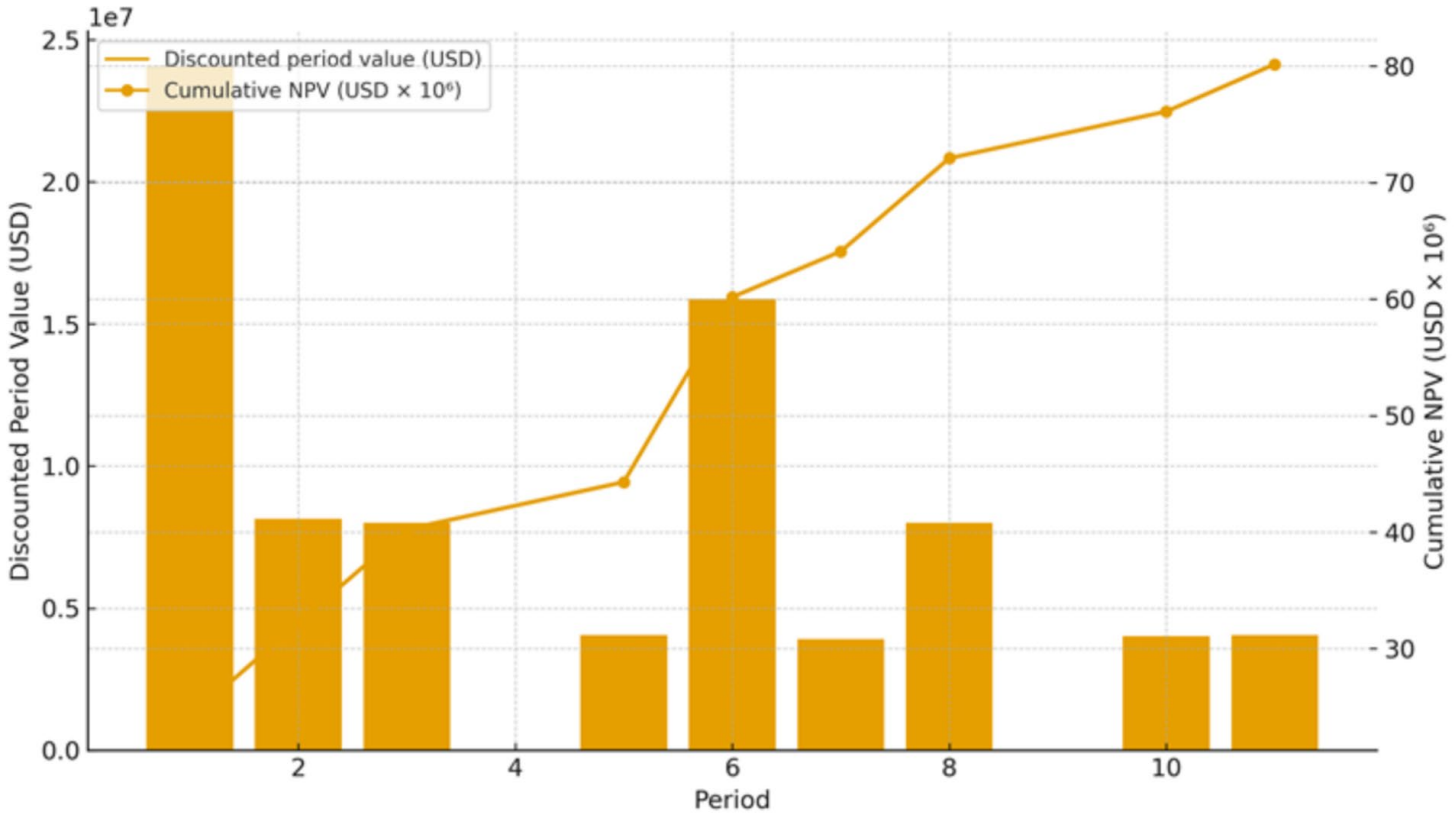
Discounted period value (bars, USD) and cumulative NPV (line, USD) over the 12-period horizon; discount rate = 8%.

**Figure 5:**
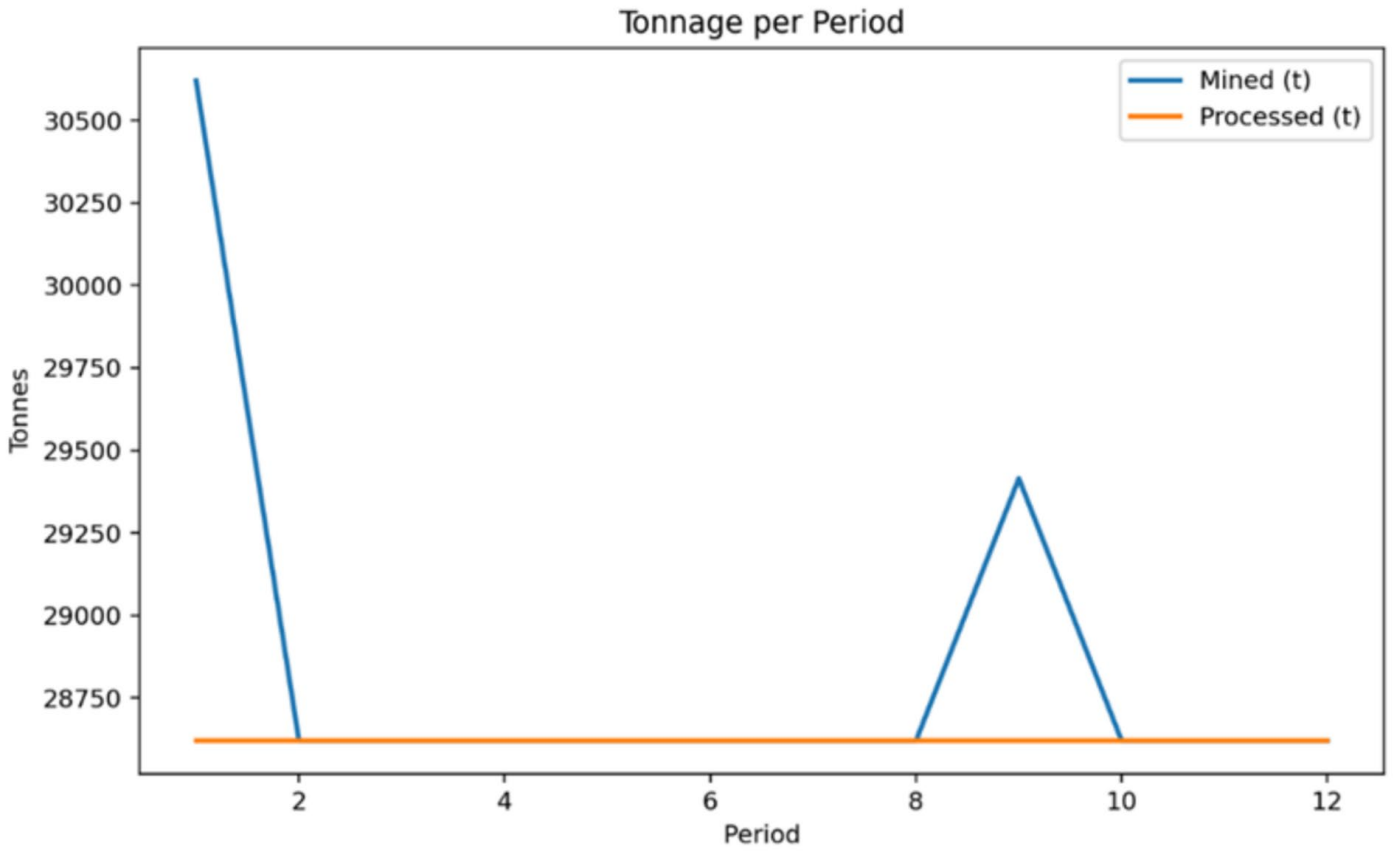
Mined and processed tonnage per period for the optimized schedule.

The enhanced block model contained 602 blocks defined by tonnage, grade, recovery, costs, and environmental penalties. Solving the model over a 12-period horizon with a slope angle of 55°, mining capacity of 10% of total tonnage per period, and processing capacity of 6% produced an Optimal solution. A 3D view with Period 12 emphasized is shown in Figure 3, illustrating the spatial distribution of scheduled blocks and the pit geometry preserved by the cone-based precedence rules. Figures 4, 5 show the discounted period value and cumulative NPV across the planning horizon and processed tonnage per period for the optimized schedule, respectively.

**Figure 6:**
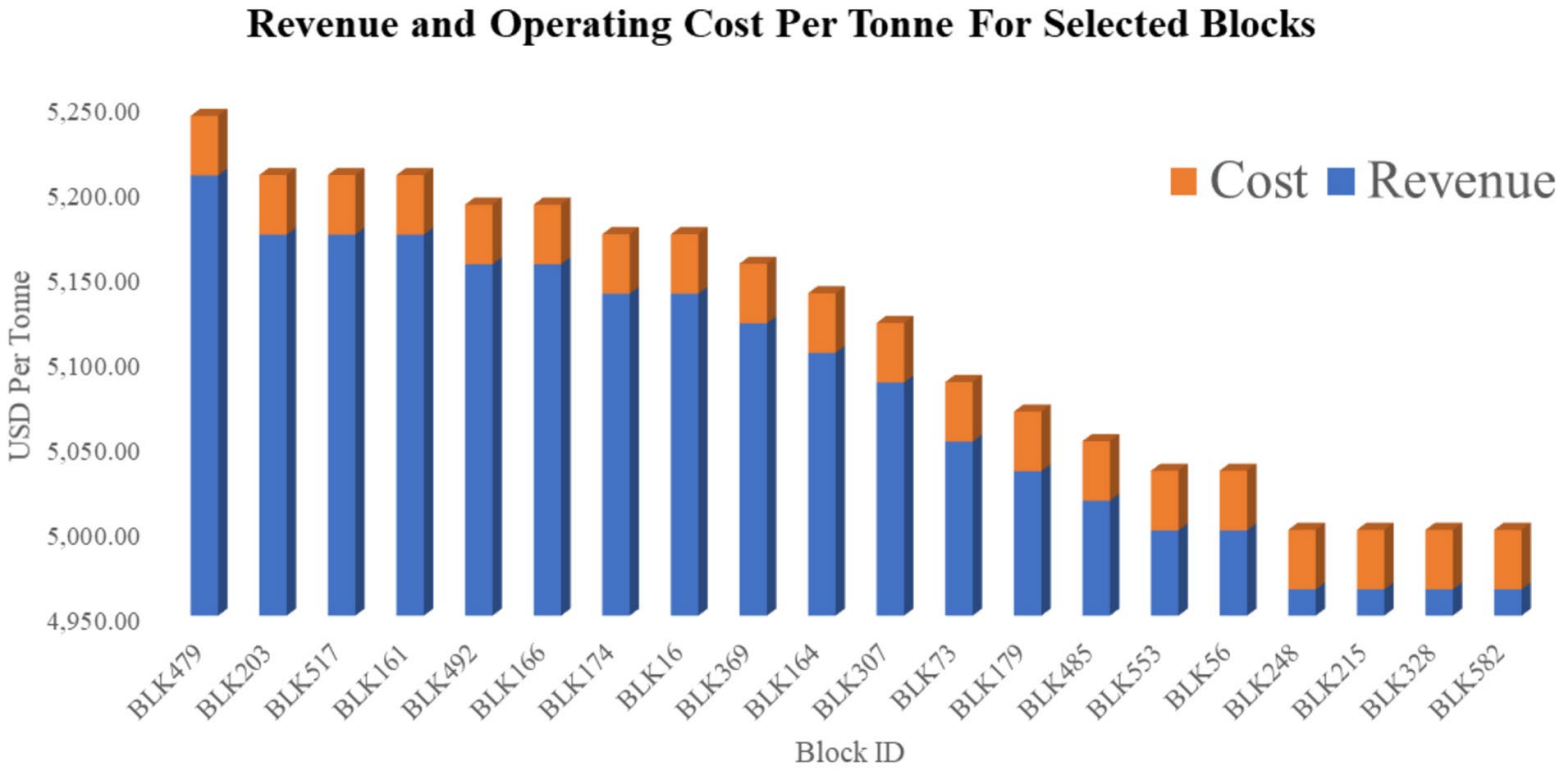
Revenue versus operating cost for selected high-value blocks.

The plan scheduled 435 blocks (≈72% of the model) and achieved a discounted NPV of USD 674.83 million. The optimized schedule showed a strong preference for mining high-value ore blocks in early periods. This front-loading of revenues is consistent with patterns reported in previous scheduling studies, which show that optimized extraction sequences tend to prioritize high-grade ore when operational and slope constraints permit. In the present case, all precedence rules were respected, with no block mined before its overlying neighbors, confirming that the three-dimensional cone-precedence matrix operated correctly. Production volumes also stayed within capacity limits each period, showing that the solution is not only mathematically optimal but also operationally feasible. Table 7 and Figure 6 show a representative sample of the first 20 scheduled blocks, ordered by value; values reflect revenue, costs, and environmental penalty, and revenue and operating costs for the top-value-contributing scheduled blocks, respectively.

**Table 7:** Sample scheduled blocks.

Block ID	Period	Grade (g/t)	Tonnes	Revenue Per Tonne (USD)	Cost Per Tonne (USD)	Penalty Per Tonne (USD)	Value (USD)
BLK479	1	3	795	5,208.42	35	3	4,110,481.56
BLK203	2	2.98	795	5,173.69	35	3	4,082,876.95
BLK517	1	2.98	795	5,173.69	35	3	4,082,876.95
BLK161	8	2.98	795	5,173.69	35	3	4,082,876.95
BLK492	1	2.97	795	5,156.33	35	3	4,069,074.65
BLK166	2	2.97	795	5,156.33	35	3	4,069,074.65
BLK174	5	2.96	795	5,138.97	35	3	4,055,272.34
BLK16	11	2.96	795	5,138.97	35	3	4,055,272.34
BLK369	3	2.95	795	5,121.61	35	3	4,041,470.04
BLK164	6	2.94	795	5,104.25	35	3	4,027,667.73
BLK307	10	2.93	795	5,086.89	35	3	4,013,865.43
BLK73	6	2.91	795	5,052.16	35	3	3,986,260.81
BLK179	3	2.9	795	5,034.80	35	3	3,972,458.51
BLK485	1	2.89	795	5,017.44	35	3	3,958,656.20
BLK553	1	2.88	795	5,000.08	35	3	3,944,853.90
BLK56	6	2.88	795	5,000.08	35	3	3,944,853.90
BLK248	7	2.86	795	4,965.36	35	3	3,917,249.29
BLK215	8	2.86	795	4,965.36	35	3	3,917,249.29
BLK328	6	2.86	795	4,965.36	35	3	3,917,249.29
BLK582	1	2.86	795	4,965.36	35	3	3,917,249.29

For comparative interpretation, four conceptual scheduling scenarios were evaluated to illustrate the incremental effect of environmental penalization: (i) Base case – model run without penalizations; (ii) Base case + penalty adjustment – same schedule, but penalized values applied post-optimization; (iii) Optimized case – model re-run including penalizations in the objective; and (iv) Optimized + penalized case – evaluation of the optimized schedule including environmental costs. These comparative cases highlight how the inclusion of environmental penalties influences extraction order, profitability, and sustainability alignment.

Preliminary sensitivity checks indicated that introducing environmental penalties reduced total NPV by approximately 3–5%, consistent with the magnitude reported in earlier ESG-integrated optimization studies (Xu et al., 2018; Aghdamigargari et al., 2024). This confirms that the penalization level meaningfully adjusts block prioritization without overwhelming the economic objective, thereby balancing profitability and sustainability considerations within realistic bounds. The results also illustrate the importance of distinguishing between block value and project NPV. While block value is computed as revenue minus mining, processing, and environmental costs, NPV is only realized after blocks are sequenced into specific periods and discounted. This prevents the misinterpretation that a block holds NPV prior to scheduling. Similar clarification has been emphasized in previous work, where block values are used as inputs but NPV emerges only after the extraction sequence is established (Das et al., 2022). Blocks with high grades but significant environmental penalties were systematically excluded from the schedule, confirming that embedding sustainability costs directly into block valuations can alter extraction priorities. Prior penalty-based scheduling research has shown that including penalty terms and dynamic cut-offs shifts schedules toward more environmentally responsible extraction sequences (Lotfi, 2024). The present model reflects the same behavior, filtering out blocks whose profitability diminishes once environmental costs are incorporated.

The baseline solution confirms that an open-source Python implementation can deliver competitive results. Comparable levels of feasibility and economic performance have been reported in recent scheduling studies that combine integer programming and heuristic methods (Alipour et al., 2022; Levinson and Dimitrakopoulos, 2024).

## 4 Discussion

The results demonstrate that the proposed open-source optimization framework can generate economically viable and operationally feasible production schedules while explicitly incorporating sustainability considerations. The model achieved a high discounted Net Present Value (NPV) under strict adherence to geotechnical precedence and capacity constraints, confirming that transparent, Python-based implementations can effectively replicate the performance of more established proprietary systems. A key insight from the results is the influence of scheduling on value realization. While block values are determined by intrinsic properties such as grade, cost, and environmental penalties, the timing of extraction governs their contribution to overall project NPV. This reinforces the importance of sequencing decisions in mine planning, where early extraction of high-value blocks significantly enhances economic outcomes due to discounting effects. The model consistently prioritized high-grade material in early periods, demonstrating behavior aligned with established optimization principles.

The integration of environmental penalties directly into the objective function proved effective in shaping extraction decisions. Blocks associated with higher environmental costs were systematically deprioritized or excluded, resulting in schedules that reflect a balance between profitability and environmental responsibility. This outcome highlights the practical value of embedding ESG considerations within optimization models rather than applying them as external adjustments. Importantly, the observed reduction in NPV under penalized scenarios remained moderate, indicating that sustainability objectives can be incorporated without substantially compromising economic performance.

Sensitivity analysis further confirmed the robustness of the framework. Variations in discount rate, mining capacity, and gold price produced predictable and interpretable impacts on scheduling outcomes. Changes in discount rate primarily affected the weighting of future revenues, while mining capacity influenced the timing of extraction without significantly altering block selection. Gold price exhibited the strongest effect, driving inclusion or exclusion of marginal blocks. Despite these variations, the model consistently maintained feasibility and stable scheduling patterns, demonstrating resilience under changing economic and operational conditions. From a methodological perspective, the adoption of the “by” (cumulative) formulation contributed to improved computational efficiency and structural clarity. By encoding precedence constraints in cumulative form, the model benefits from stronger linear relaxations, which enhance solver performance and facilitate interpretation of mining sequences. This confirms that established theoretical advances in scheduling formulations can be effectively implemented within open-source environments.

The practical implications of this work lie in its transparency and extensibility. The availability of the full codebase and dataset enables reproducibility, validation, and adaptation to different mining contexts. In addition, the integration of data processing, optimization, and visualization within a single framework supports its application as a decision-support tool for strategic mine planning. The generated outputs, such as tonnage profiles and NPV trends, provide intuitive insights that can assist planners in evaluating trade-offs between economic and environmental objectives.

Although this study is based on a 602-block synthetic model, the proposed framework is inherently scalable and designed for application to large and complex mining problems. Its modular structure and compatibility with MineLib datasets enable direct extension to benchmark instances exceeding 100,000 blocks without modification to the core model. Accordingly, this work should be viewed as a methodological validation of the proposed approach, demonstrating its feasibility, transparency, and adaptability. Future research will focus on large-scale benchmarking and real-world case studies to further evaluate computational performance and industrial applicability. In addition, the integration of stochastic elements and hybrid optimization techniques is anticipated to enhance the framework’s capability to operate under geological and economic uncertainty.

## 5 Conclusion

This study developed and implemented a fully reproducible, open-source Python-based optimization framework for open-pit production scheduling that integrates economic and environmental considerations within a unified decision-making structure. Using a mixed-integer “by” formulation with three-dimensional precedence constraints, the model successfully generated feasible and high-value production schedules while maintaining strict compliance with operational and geotechnical limits. The results demonstrate that sustainability-oriented scheduling can be achieved through direct integration of environmental penalties into block valuations, allowing economic and environmental trade-offs to be evaluated within a single optimization framework. At the same time, the distinction between block value and project-level NPV provides a more accurate representation of value realization, emphasizing the critical role of extraction sequencing in long-term mine planning. The robustness of the framework under varying economic and operational conditions further supports its applicability as a reliable decision-support tool.

Beyond its methodological contributions, the framework advances transparency and reproducibility in mine planning by providing an accessible alternative to proprietary systems. Its modular and dataset-agnostic design enables straightforward adaptation to different block models and supports future benchmarking on large-scale instances. Notwithstanding these strengths, the study is subject to limitations. The current implementation was evaluated on a medium-scale synthetic dataset, and the environmental penalty structure was simplified. Future work should therefore focus on large-scale validation using benchmark datasets such as MineLib, integration of stochastic elements to capture geological and economic uncertainty, and expansion of the penalty framework to incorporate broader sustainability indicators.

Overall, this work establishes a practical and extensible foundation for sustainability-integrated mine scheduling, demonstrating how open-source optimization can support more transparent, adaptable, and responsible decision-making in modern mining operations.

## Data Availability

The datasets presented in this study can be found in online repositories. The names of the repository/repositories and accession number(s) can be found in the article/supplementary material.
